# A cross linguistic study on orthographic influence during auditory word recognition

**DOI:** 10.1038/s41598-025-92885-x

**Published:** 2025-03-11

**Authors:** Alberto Furgoni, Clara D. Martin, Antje Stoehr

**Affiliations:** 1 Basque Center on Cognition, Brain and Language, Paseo Mikeletegi 69, Donostia-San Sebastián, 20009 Spain; 2 Ikerbasque - Basque Foundation for Science, Bilbao, Spain

**Keywords:** Auditory word processing, Orthographic consistency effect, Orthographic depth, French, Spanish, Auditory lexical decision task, Language, Psychology

## Abstract

Learning to read affects speech perception. For example, the ability of listeners to recognize consistently spelled words faster than inconsistently spelled words is a robust finding called the Orthographic Consistency Effect (OCE). Previous studies located the OCE at the rime level and focused on languages with opaque orthographies. This study investigates whether the OCE also emerges at the phonemic level and is a general phenomenon of languages with alphabetic scripts, including those with transparent writing systems. Thirty French (opaque language) and 30 Spanish (transparent language) listeners participated in an auditory lexical decision task featuring words and pseudowords including either only consistently spelled phonemes or also inconsistently spelled phonemes. Our results revealed an OCE in both French and Spanish which surfaced as longer reaction times in response to inconsistently spelled words and pseudowords. However, when analyzing the data split by language, the OCE was only detectable in French but not in Spanish. Our findings have two theoretical implications. First, they show that auditory lexical processing is impacted by orthographic information that is retrieved at the phonemic level, not just the rime level. Second, they suggest that the OCE may be modulated by a language’s opacity. In conclusion, our study highlights the depth of literacy effects on auditory language processing and calls for further investigations involving highly transparent languages.

## Introduction

Reading and writing are among the most basic cognitive skills of literate people. Interestingly, learning to read and write influences speech processing. For instance, research into orthographic consistency effects has shown that consistent (i.e., unambiguous) sound-to-spelling mappings impose less cognitive effort in word recognition and processing compared with inconsistent (i.e., ambiguous) mappings, a phenomenon known as the Orthographic Consistency Effect (OCE)^1–9^. The OCE highlights the interplay between written and spoken language, illustrating how predictable orthographic patterns facilitate quicker and more reliable phonological retrieval. In one of the first studies on the OCE, Seidenberg and Tanenhaus^10^ demonstrated that native English listeners were faster to identify auditorily presented word pairs as rhyming when they matched in spelling (e.g., *globe* and *probe*) than when they mismatched in spelling (e.g., *rye* and *tie*). The robust finding that inconsistent sound-to-spelling mappings interfere in auditory word processing suggests that the orthographic representation of a word is automatically accessed during this process. Understanding the OCE is crucial for insights into reading fluency, language acquisition, and the cognitive processes underlying literacy. It also has practical implications for educational strategies and interventions aimed at improving reading skills and addressing reading difficulties.

Building on earlier studies, on orthographic effects in the auditory modality^10–12^, Ziegler and Ferrand^9^ employed an auditory lexical decision task to investigate whether words with inconsistently spelled rimes are cognitively costlier to process than words with consistently spelled rimes. The advantage of auditory lexical decision tasks is that they directly test lexical access and, unlike the word pair rhyming task, do not require a prime or a cue in order to elicit a potential OCE. In their study, French listeners were quicker and more accurate at recognizing words with consistently spelled rather than inconsistently spelled rimes, confirming that orthography influences auditory word processing.

There are different hypotheses on whether the OCE is a pre-lexical or a post-lexical effect. On the one hand, Ventura and colleagues^4^ argued in favor of the post-lexical nature of the OCE. They claimed that lexical access is necessary for the OCE to occur, as they observed no OCE in a shadowing task (which elicits online processes) but only in a lexical decision task. However, it is important to note that separating perception and production processes in the shadowing task is challenging^13^. On the other hand, Petrova and colleagues^14^ argued that the OCE is an online process because rime detection tasks inherently require word segmentation. Additionally, they found no significant difference in the magnitude of the OCE between the rime detection and lexical decision tasks. More consistent evidence on the pre-lexical nature of the OCE comes from studies with electrophysiological data, as the effect was observed in a time window related to pre-lexical processing (i.e., < 400ms) when participants were carrying out non-metaphonological tasks (e.g., semantic or lexical decision)^15–17^. Even though there is consistent evidence in favor of the pre-lexical nature of the OCE, it remains unclear why this effect is not consistently found in pseudoword processing. As discussed later, the present study intends to shed light on pseudoword processing starting from the assumption that the OCE is a pre-lexical effect.

The use of a lexical decision task enabled Ziegler and Ferrand^9^ to also test for an OCE in pseudoword processing. Although they found no evidence of an effect, the OCE was found in pseudoword processing in a later study, again with French listeners, by Pattamadilok and colleagues^2^. Considering that pseudowords are necessarily decoded phonemically, it is surprising that the evidence for an OCE in pseudoword processing is weak. Two arguments have been put forward to explain this finding^9^: First, pseudowords do not have lexical spellings and therefore they cannot be as (in)consistent as real words.

Second, because lexical decision is a classification task, pseudoword stimulus items may be “timed out” when they do not reach an activation threshold, which is why they do not show some of the effects that are typically seen for words.

Another possible explanation may be that the OCE does not affect the phonemic level during lexical access but, to our knowledge, no study has investigated if the OCE in auditory word recognition operates at the phonemic level (i.e., an inconsistent phoneme can be spelled in various ways, such as French /f/ = < f > or < ph > ) as well as the rime level. Support for a phoneme-level OCE is provided by studies using visual word recognition tasks, writing tasks, and metalinguistic tasks such as phoneme deletion, which have found orthographic effects on the phonemic level. For example, in a study with French native speakers, although no evidence for a phoneme-level OCE was found in a visual lexical decision task, an OCE was elicited by a writing task measuring writing latencies and error rates^18^. Moreover, in a later study using a lexical decision task, English native speakers recognized printed words containing only consistent phoneme-to-grapheme mappings more quickly and accurately than words containing inconsistent phoneme-to-grapheme mappings^19^. Metalinguistic tasks demonstrate that English native speakers manipulate individual consistently spelled phonemes more effectively than inconsistently spelled phonemes^20^. Similarly, English native speakers recognize consistently spelled phonemes more accurately and faster than inconsistently spelled phonemes in word-initial position^21^. In sum, evidence suggests that orthographic (in)consistency affects processing at the phoneme level, but it remains untested whether phoneme-level orthographic inconsistencies affect auditory word recognition.

Another important issue that has not been thoroughly addressed by previous research is whether and to what extent speakers of transparent languages with few inconsistencies are sensitive to the rare irregularities in their spelling system during auditory lexical processing. So far, Portuguese is the only language with fairly transparent phoneme-to-grapheme mappings for which the OCE has been tested and demonstrated^4,5^. However, Portuguese still has a relatively rich phonological inventory and comprises considerably more inconsistencies than, for example, Spanish. The difference in orthographic transparency between Portuguese and Spanish becomes evident when comparing children who are learning to read in the two languages: Between the first and fourth grades (i.e., between six to ten years of age), Spanish children make fewer phonological errors during reading than grade-matched Portuguese children^22^. Thus, the present study explores the OCE in a highly transparent language, namely Spanish.

Finally, if auditory lexical processing in transparent languages is affected by the OCE, are there any cross-linguistic differences in the OCE in alphabetic writing systems? For instance, Pattamadilok and colleagues^2^ replicated Ventura and colleagues’^4^ findings on the OCE in Portuguese using French as the target language, with both studies finding the OCE in an auditory lexical decision task but not in a shadowing task. Yet, the OCE affected both word and pseudoword processing in French, while it only affected word processing in Portuguese^4^. Taken together, these diverging results suggest that native listeners of French– an opaque language – show more interference from (inconsistent) orthography in auditory lexical processing than native listeners of Portuguese – a more transparent language – which could explain why an OCE was also observed in French pseudowords. Pattamadilok and colleagues^2^ argued that French listeners rely more on orthographic representations in lexical processing because they help with the selection of the correct lexical entry (in French, /pɛ̃/ can refer to *pain* ‘bread’, *pin* ‘pine’, *peins* ‘(you) paint’, etc.). Alternatively, the link between phonology and orthography might be stronger in French than in Portuguese because of different orthographic transparency and its implications in reading acquisition:

French-speaking children need to rely on sound-to-spelling associations more than Portuguese-speaking children when they learn to read. This could also explain why the OCE was observed in pseudoword processing in French. It should be noted that French and Portuguese were compared at the rime level, and in two separate studies, which complicates a direct comparison. Building on the initial insights from these studies^2,4^, here we conduct a more systematic cross-linguistic comparison to understand how the OCE affects languages.

(1) at the phonemic level and (2) depending on the opacity of the spelling system.

### The present study

The present study investigates whether the OCE emerges at the phonemic level and whether it is also present in Spanish, an orthographically transparent language. Specifically, we asked the following two research questions:

1. Does the OCE emerge at the phonemic level? In the auditory modality, inconsistencies are not limited to rime units, but are also present at the phonemic level: A single phoneme can often be represented by multiple graphemes in writing (e.g., /f/ in French can be spelled either < f > or < ph> ). We predict that a word or pseudoword containing only consistent phonemes should be recognized faster than one containing also inconsistent phonemes, given that in the former instance, each phoneme co-activates only one orthographic representation (e.g., the French pseudoword /bun/ can only activate < boune> ). Conversely, a word or a pseudoword containing at least some inconsistent phonemes should lead to costlier processing, surfacing as longer reaction times, if inconsistent phonemes co-activate multiple orthographic representations (e.g., the French pseudoword /fub/ could activate both < foube > and < phoube> ).
2. Does the OCE influence auditory word processing in a transparent language like Spanish similarly to how it impacts auditory word processing in an opaque language like French? Considering that the literature on the OCE is largely based on studies using opaque languages, a direct comparison of the OCE elicited in transparent vs. opaque languages can reveal if it is generalizable to a wider range of alphabetic languages. Based on findings from Portuguese, the most transparent language studied so far, we predict that the OCE will be apparent in Spanish, even though it contains only a few inconsistent phoneme-to-grapheme mappings. Thus, we hypothesize that even in highly transparent languages, inconsistent phonemes elicit processing costs, surfacing as longer reaction times.

To answer these questions, native listeners of French (a highly opaque language) and Spanish (a transparent language) participated in an auditory lexical decision task in French and Spanish, respectively. The use of an auditory lexical decision task was motivated by earlier research, which suggests that this paradigm can elicit an OCE without employing any prime or cue^9^. Spanish and French are suitable for a cross-linguistic comparison because they both display a simple syllabic complexity^23^, allowing us to focus on the phonemic level.

## Methodology

### Participants

Thirty native French listeners (17 female, *M*_age_= 21.83 years, *SD*_age_=2.13) and 30 native Spanish listeners (17 female, *M*_age_= 24.23 years, *SD*_age_=3.11) participated in the study. All participants had university-level education.

Based on self-report, participants of both groups had been exposed to their native language since birth and they all predominantly used their native language in everyday life. No specific learning impairments and no hearing or uncorrected vision problems were reported. French and Spanish participants were matched on non-verbal IQ (*p* = .371), as measured by the Kaufman Brief Intelligence Test^24^. The French cohort was recruited from the University of Bordeaux (France); Spanish participants were recruited from the participant pool of the Basque Center on Cognition, Brain and Language (Spain). The experiment was approved by the Basque Center on Cognition, Brain and Language’s Ethics Committee [approval number 031218D] and performed in accordance with the Declaration of Helsinki. This ethics approval was also valid at the University of Bordeaux and no additional ethics approval was required. Participants provided written informed consent prior to starting the experiment and received monetary compensation for their participation.

### Materials

All materials and information on stimulus matching are available on the OSF. Stimuli were recorded by male native speakers of French and Spanish in a sound-attenuating chamber using a Marantz PMD 671 digital recorder with a Sennheiser ME65 microphone and were digitized at 44.1 kHz. All stimuli were scaled to 65 dB and a 50 ms interval of silence was added to the beginning of each audio file to allow for sufficient loading time in the experimental software. The stimulus list for each language consisted of 60 words and 60 pseudowords. For each lexicality condition (words; pseudowords), 50% of the stimuli contained phonemes with only one possible spelling (hereafter, consistent items). In the other 50% of the stimuli, at least the first phoneme in each item could be spelled in more than one way (hereafter, inconsistent items). Inconsistent French items contained up to five inconsistent phonemes, whereas Spanish items contained up to three. The discrepancy between the two languages was due to differences in the opacity of French and Spanish: French words commonly include several inconsistent phonemes; Spanish is a transparent language and has fewer words with multiple inconsistent phonemes, especially when word frequency and length are controlled. All inconsistent phonemes were consonants.

Pseudowords were created from the word lists by changing at least the consonants at the beginning of each syllable, respecting phonotactic constraints (Table 1).

**Table 1:** Stimulus examples for French and Spanish.

	French		Spanish	
	Consistent	Inconsistent	Consistent	Inconsistent
Words	porte/pɔʁt/	flaque/**f**la**k**/	fruta/fruta/	brazo/**b**ɾa**θ**o/
Pseudowords	/lɔʁv/	/**s**la**s**/	/lursa/	/**k**ra**x**o/

Note. Inconsistent phonemes highlighted in bold.

Overall, consistent and inconsistent words within each language were matched on a series of potentially confounding variables such as word frequency, phonological and orthographic length, bigram and biphone frequency, first phoneme frequency, neighborhood density, and their uniqueness point within each language. Cross-linguistically, words were matched on frequency (Zipf’s log frequency), number of letters and the duration of the audio recordings. These variables were retrieved from the Lexique database^25^ and from the EsPal database^26^ for French and Spanish, respectively. The phonological and orthographic neighborhood densities of words and pseudowords of both languages were determined with CLEARPOND^27^. Consistent and inconsistent pseudowords were matched on these variables within languages. Across languages, pseudowords were matched on mean phonological neighborhood.

### Apparatus and procedure

The experiment was administered on an HP EliteBook Folio 1040 G3 laptop computer using OpenSesame software (version 3.2.4^28^). Auditory stimuli were presented over Sennheiser GSP 350 headphones. The experiment was run in sound-attenuating chambers at the University of Bordeaux and at the Basque Center on Cognition, Brain and Language’s satellite laboratory at the University of the Basque Country in Donostia-San Sebastián.

Participants were tested on an auditory lexical decision task, in which they had to respond via key press whether each stimulus item was a real word or not. Participants were instructed to respond as quickly and as accurately as possible. The respective keys were labeled on the keyboard; half of the participants pressed the left key for words and the other half pressed the right key for words. Before starting the main part of the experiment, the participants completed a practice phase with 10 extra items (5 words, 5 pseudowords). The participants only received feedback on their responses in the practice phase.

During both the practice phase and the main experiment, each trial started with a fixation point appearing in the center of the screen for 500 ms, which was followed by the auditory stimulus. Reaction time measurement started at stimulus onset and ended with the key press. After participants gave their response, the next trial was automatically initiated. The items were presented in randomized order across participants. The task lasted approximately 15 min.

### Data analyses

Data and analysis scripts are available on the OSF. We conducted the statistical data analyses in RStudio (version 2023.12.1.402^29^) run on R (version 4.2.0^30^) and using the lme4 package (version 1.1–29^31^). We obtained *p* values for *t* statistics through the lmerTest package (version 3.1-3^32^). We employed an alpha level of 0.05 for interpreting statistical significance. We inverse-transformed reaction times as indicated by the Box–Cox transformation^33^ performed using the MASS package (version 7.3–57^34^), and removed data points with standardized residuals more than 2.5 standard deviations from 0 as outliers using the LMERConvenienceFunctions package^35^. We ran a linear mixed-effects model with *inverse-transformed reaction times* as the dependent variable. The model had deviation coded fixed effects for *Consistency* (consistent=-0.5; inconsistent = 0.5), *Lexicality* (word=-0.5; pseudoword = 0.5), and *Language* (French=-0.5; Spanish = 0.5) with an interaction term including lower-level interactions. The model had random intercepts for *Participant* and *Item* and by-*Participant* random slopes for *Consistency* and *Lexicality*. Following Ventura and colleagues^5^, we ran one model including both words and pseudowords.

Due to technical problems, two Spanish participants did not fully complete the task, resulting in the loss of 39 data points. The two French consistent pseudowords **/**myv/ and.

/sadʁ/ were excluded from the analyses because they elicited accuracy rates below chance (1.68% of the entire dataset). The remaining consistent and inconsistent pseudowords remained matched on all confounding variables.

## Results

**Fig. 1:**
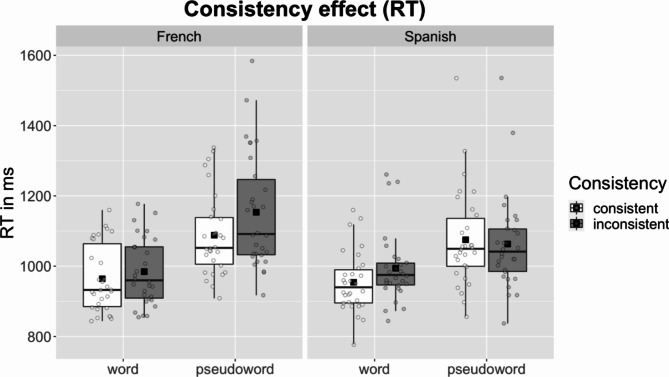
Raw reaction times (RT) by Consistency and Lexicality in French and Spanish. Note. Each dot shows an individual participant and the black square shows the group mean.

Reaction time analyses were conducted on correctly answered trials (6,840 trials; 96.32% of the data). An additional 147 data points were removed as outliers (2.15% of the data). The model detected significant effects of *Consistency* and *Lexicality*, showing that participants took longer to respond to inconsistent items than to consistent items and to pseudowords than to words (Fig. 1; Table 2). No other significant effects or interactions were detected (We ran an additional model in which we excluded French trials with 4 and 5 inconsistencies to ensure that these items are not driving the observed effects. This model likewise observed effects for Consistency (*Estimate*=-2.475e-05, *SE* = 1.068e-05, *t*=-2.318, *p* = .0213) and Lexicality (*Estimate*=-1.040e-04, *SE* = 1.255e-05, *t*=-8.282, *p* < .001) and no other significant main effects or interactions.).

**Table 2:** Results table of the reaction time analysis.

	Estimate	SE	t	*p*
(Intercept)	1.001e-03	1.285e-05	77.913	< 0.001
Consistency	-2.590e-05	1.055e-05	-2.454	0.015
Language	1.985e-05	2.570e-05	0.773	0.442
Lexicality	-1.057e-04	1.238e-05	-8.536	< 0.001
Consistency × Language	1.734e-05	2.111e-05	0.822	0.412
Consistency × Lexicality	1.296e-05	2.062e-05	0.629	0.530
Language × Lexicality	4.589e-05	2.477e-05	1.853	0.065
Consistency × Language × Lexicality	5.817e-05	4.124e.-5	1.411	0.160

To further explore the data, we split the data by language and ran two separate linear-mixed effect models to see if the OCE was detectable in each language individually. Each model had the same structure of the main model except for having Language as an independent variable. The model run on the French data showed main effects of Consistency and Lexicality with no interactions (Table 3) while the model run on the Spanish data showed only a main effect for Lexicality (Table 4).

**Table 3:** Results table of the reaction time analysis for the French data.

	Estimate	SE	t	*p*
(Intercept)	9.917e-04	1.704e-05	58.205	< 0.001
Consistency	-3.492e-05	1.271e-05	-2.748	0.007
Lexicality	-1.159e-04	1.606e-05	-7.219	< 0.001
Consistency × Lexicality	-8.467e-08	2.421e-05	-0.003	0.997

**Table 4:** Results table of the reaction time analysis for the Spanish data.

	Estimate	SE	t	*p*
(Intercept)	1.010e-03	1.896e-05	53.266	< 0.001
Consistency	-1.652e-05	1.579e-05	-1.047	0.297
Lexicality	-8.365e-05	1.844e-05	-4.536	< 0.001
Consistency × Lexicality	4.246e-05	3.074e-05	1.381	0.170

## Discussion

The Orthographic Consistency Effect (OCE) is the processing advantage of words with a consistent spelling, which has previously been observed in words with consistent compared to inconsistent rime spellings in orthographically opaque languages (e.g., *globe* and *probe* are judged as rhyming words faster than rye and tie^10^). In contrast to previous research, we manipulated orthographic inconsistency at the phonemic rather than the suprasegmental rime level. This manipulation resulted in words and pseudowords containing phonemes that either mapped onto only a single orthographic representation (consistent words/pseudowords) and words and pseudowords containing some phonemes with multiple potential spellings (inconsistent words/pseudowords). Our results indicate that this phonemic manipulation also reveals an OCE in auditory processing of both words and pseudowords, yet with differences between French and Spanish.

Reaction time analyses showed that inconsistencies at the phonemic level impacted auditory lexical processing: native French listeners were slower when identifying inconsistent compared to consistent items. This novel finding informs previous research that investigated the OCE at the rime or syllabic levels^4,7,9,36^.

For example, the OCE found in Ziegler and Ferrand^9^ was attributed to the (in)consistencies of the sound-to-spelling mappings of French rimes (e.g., *gamme* /gam/ is inconsistent because -/am/ can be spelled -< am>, -< ame > or -< amme> ) while the individual phonemes constituting the rime (/a/ and /m/ in /gam/) are consistent when considered individually. Yet, some stimuli like *gaffe* /gaf/ are inconsistent only because one phoneme (/f/ in /gaf/) in the rime can be spelled in multiple ways (< f > or < ph > in *gaffe*). It is consequently not possible to give a univocal explanation of the linguistic level affected by the OCE. Our study disentangles this ambiguity by showing that (in)consistencies of individual phonemes can affect auditory lexical processing.

Furthermore, our findings extend previous research that showed a processing disadvantage for words containing inconsistently spelled phonemes in word writing^19^ and visual word recognition^20^ as well as previous research on metalinguistic skills showing that listeners more easily manipulate consistent than inconsistent phonemes^21^ and that they recognize consistent phonemes faster and more accurately than inconsistent phonemes^22^. This implies, therefore, that auditory lexical processing is affected not only by rime (in)consistency but also by phonemic (in)consistency. This is only logical, given that the phonemic level plays a unique role in language processing in general for people who are literate in alphabetical languages, as demonstrated by the finding that phonemic awareness is a better predictor of success in learning to read than onset/rime awareness^37^. Since literacy derives from establishing a relationship between individual phonemes and their orthographic representations, our findings confirm the importance of literacy in auditory language processing. In fact, it has recently been argued^38^ that phonemes can be regarded as a by-product of literacy as their relation with their orthographic representations strongly impacts auditory language processing in literate people.

Previous research investigated the OCE mainly in languages with opaque orthographies (i.e., English and French) or in languages with a fairly transparent orthography (i.e., Portuguese). Our study shows that orthographic (in)consistency does not detectably interact with language, suggesting that the OCE affected both French and Spanish. It thus seems that the OCE can be detected in a broader spectrum of alphabetical languages ranging from the least to the most transparent. However, when splitting the data by language, no OCE was detectable in the Spanish data alone. Therefore, our results seem to be more inconclusive in Spanish, a highly transparent language, where only a numerical tendency appeared for an OCE in auditory word processing. As the OCE seems to be prominent in auditory language processing when the language is opaque rather than transparent, more research into the OCE in transparent languages is needed to attest to what extent the OCE affects transparent languages.

Interestingly, our results revealed an OCE in both word and pseudoword processing, indicating that native French listeners strategically rely on phoneme-to-grapheme conversions during pseudoword recognition. This is remarkable as the potential top-down processes of word recognition cannot have the same impact on pseudoword recognition because their processing is independent of lexical access or partial lexical activation^39^.

However, it is possible that the underlying mechanisms of the OCE for words and pseudowords differ, given that real words have a representation in long-term memory while pseudowords do not. Consequently, even if both types of stimuli triggered apparently similar consistency effects, they may not relate to the same underlying processes. The OCE observed for real words could be related to comparisons between phonological memory traces in working memory to orthographic memory traces in long-term memory, while the OCE observed in pseudowords could be related to phoneme-to-grapheme conversion effects. Our results on the OCE in words and pseudowords are in line with Pattamadilok and colleagues^2^ but in contrast with other studies on the OCE in French^7,9^ and Portuguese^4^, which did not find an OCE in pseudoword recognition. An interpretation of this discrepancy could be that the competition between different orthographic representations of an inconsistent phoneme is greater than the competition between different orthographic representations of an inconsistent rime at the pre-lexical stage of auditory language processing.

In other words, the difference between the findings on French pseudoword recognition in this study and the lack of an effect in French pseudoword recognition in other studies suggests that inconsistencies at the phonemic level are more impactful than inconsistencies at the rime level, at least with inconsistent phonemes in initial position in the word/pseudoword. Word onset playing a major role in auditory word recognition, the impact of inconsistencies in word initial position might be larger. Future studies should explore this question by comparing the magnitude of the OCE with inconsistent phonemes being in initial or final position.

In conclusion, the present study showed that native listeners of opaque languages are affected by orthographic inconsistencies at the phonemic level during auditory word and pseudoword processing. Future research should compare the magnitude of the OCE at the rime and phonemic levels in opaque and transparent languages to disentangle how orthographic (in)consistencies at different linguistic levels impact auditory word recognition.

## Data Availability

Data and analysis code are available on the OSF: https://osf.io/xd4vh/?view_only=ad18da42e4d24eb98974f08be05e8cd9.
